# Life-threatening complication due to double-J stent: renal subcapsular hematoma

**DOI:** 10.1093/jscr/rjac329

**Published:** 2022-07-30

**Authors:** Moez Rahoui, Yassine Ouanes, Kays Chaker, Kheireddine Mrad Dali, Mokhtar Bibi, Ahmed Sellami, Sami Ben Rhouma, Yassine Nouira

**Affiliations:** Urology Department, La Rabta Hospital, Tunis, Tunisia; Urology Department, La Rabta Hospital, Tunis, Tunisia; Urology Department, La Rabta Hospital, Tunis, Tunisia; Urology Department, La Rabta Hospital, Tunis, Tunisia; Urology Department, La Rabta Hospital, Tunis, Tunisia; Urology Department, La Rabta Hospital, Tunis, Tunisia; Urology Department, La Rabta Hospital, Tunis, Tunisia; Urology Department, La Rabta Hospital, Tunis, Tunisia

**Keywords:** double-J stent, kidney, hematoma, nephrectomy

## Abstract

The double-J ureteral stent is a standard procedure in daily urological practice. Although considered as safe, this approach is fraught with several complications. These complications are of limited severity and resolve with symptomatic treatment. In some cases, serious and life-threatening complications, such as infection and subcapsular hematoma, can occur. In the literature, a few cases of subcapsular renal hematoma secondary to ureteral stent insertion have been reported. Herein, we report a case of renal subcapsular hematoma combined with hemorrhagic shock in a 67-year-old patient who had a ureteral stent insertion one month ago.

## INTRODUCTION

The double-J (DJ) ureteral stent is a standard procedure in daily urological practice [1]. It is indicated to relieve a ureteral obstruction or as part of other endourological procedures [1]. However, there has been a worrying increase in possible complications associated with stent insertion, such as encrustation and misplacement, infections and subcapsular hematoma [2]. In the literature, a few cases of subcapsular renal hematoma secondary to ureteral stent insertion have been reported. Herein, we report a case of renal subcapsular hematoma combined with hemorrhagic shock in a 67-year-old patient who had a ureteral stent insertion 1 month ago.

## CASE REPORT

**Figure 1:**
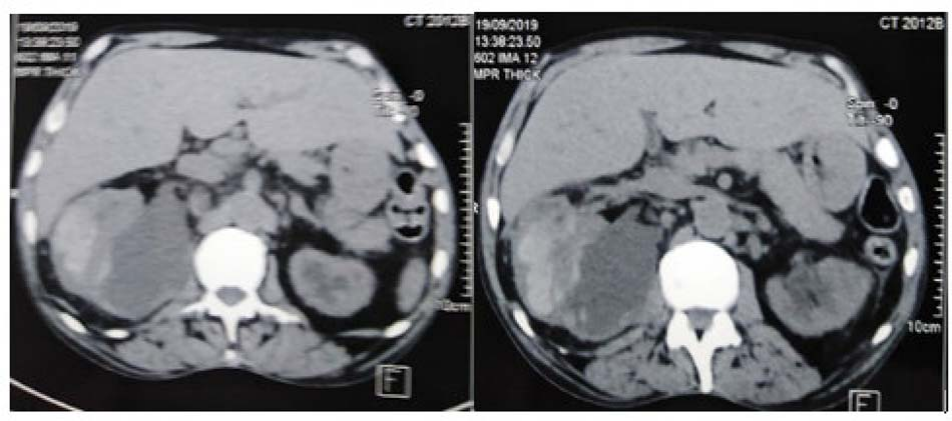
The CT scan showed a huge subcapsular hematoma (12 × 14 × 10 cm) of the right kidney.

A 67-year-old patient, smoker, diabetic on insulin, who was initially admitted to hospital for the management of a right distal ureteral stone of 1 cm. He had a right ureteroscopy with the insertion of a DJ ureteral stent. The post-operative course was uneventful. After 1 month, the patient consulted us for deterioration of the general condition associated with right lumbar pain. Clinical examination showed poor general condition, pallor, hypotension and tachycardia. The urological examination showed a contracture of the right lumbar fossa. Biological analysis showed microcytic hypochromic anemia, (hemoglobin: 4.8 g/dl), thrombocytopenia and renal failure with high C-reactive protein (CRP) levels (Table 1). The patient was admitted to the intensive care unit. After transfusion and stabilization of the hemodynamic state, a radiological exploration by computed tomography (CT) was made. The CT scan revealed a huge subcapsular hematoma (12 × 14 × 10 cm) of the right kidney (Fig. 1). The proximal end of the DJ stent was in the renal pelvis. Despite resuscitation and transfusion, the patient remains hypotensive and severely tachycardic. an open right nephrectomy was performed as an emergency. The patient had an uneventful recovery and was discharged on the seventh post-operative day. Histological examination did not show a malignant lesion. After 24 months of clinical and radiological controls, there were no functional complaints or signs of lithiasis recurrence. Renal function was normal.

## DISCUSSION

Ureteral stenting is a common urological procedure that allows the drainage of the kidney without external shunting [1]. This procedure is indicated for obstructive uropathy or as part of other endourological procedures [2]. Although considered as safe, this approach is fraught with several complications. In the available literature, the complication rate secondary to ureteral stent insertion can be as high as 79.2% [2, 3]. The most common complications are irritative voiding symptoms, flank pain, suprapubic discomfort and hematuria [2]. These complications are of limited severity and usually require symptomatic treatment and resolve on the removal of the stent. On the other hand, some more serious complications may occur, such as migration or encrustation of the stent, infection, missed stents, bladder erosions and misplaced stents [1, 3]. However, a few cases of renal parenchymal perforation and hematoma formation after insertion of a DJ stent have been reported in the literature [3].

**Table 1:** Laboratory investigations

Biochemical and hematological parameters	Value
Blood type	A-positive (A+)
Hemoglobin (g/dl)	4.8
White blood cells	14 320
Platelets	68 000
Serum creatinine (μmol/l)	274
CRP (mg/l)	232
Lactate (mmol/l)	2.4
Calcium (mmol/l)	2.3
Albumin (g/l)	34

The physiological mechanisms of subcapsular renal hematoma formation are not well understood. Some authors have suggested that the intra-renal pressure generated during stent placement may cause fragility of the renal parenchyma favoring hematoma formation [3]. In other publications, even minor trauma caused by the guide wire or misplacement or migration of the ureteral stent was another contributing factor [4, 5]. The precise timing of the development of the subcapsular hematoma is not known. It is possible that the hematoma developed during or shortly after the initial stenting or late following stent migration [2, 3]. The diagnosis of this complication may be suspected clinically and confirmed by imaging. The clinical presentation includes macro- or microscopic hematuria, back pain and less commonly a palpable mass, altered general condition, signs of shock, including hypotension and tachycardia, and may sometimes be asymptomatic. Biologically, there may be severe anemia and hemostasis disorders [3, 4]. Ultrasound and CT scans confirm the diagnosis of this complication in the majority of cases [2, 5]. In our case, the patient consulted for low back pain associated with altered general condition and signs of shock. Laboratory analysis showed severe anemia and thrombocytopenia. The diagnosis of renal subcapsular hematoma was confirmed by a CT scan.

Given the rarity of this condition, its management is not codified. It depends on the patient’s condition and the size of the hematoma [2, 4]. In some cases, the evolution is favorable by the spontaneous resorption of the hematoma [2, 3]. In other cases, treatment is based on analgesics, antibiotics and percutaneous drainage of the hematoma associated with resuscitation measures if signs of hemorrhagic shock are present [4, 5]. A stent change will be done in case of misplacement or migration [3, 5]. Our patient had a large subscapular hematoma associated with hemorrhagic shock resistant to resuscitation measures. He had an open nephrectomy with a good clinical outcome.

## CONCLUSION

Although considered as a common and safe procedure, urethral stent insertion can be associated with several complications. Clinical and radiological monitoring is recommended in order to diagnose and treat these complications promptly.

## References

[1] Pansota MS , Rasool M, Saleem MS, Tabassum SA, Hussain A. Indications and complications of double-J ureteral stenting our experience. Gomal J Med Sci 2013;11:8–12.

[2] Nomikos MS , Chousianitis Z, Georgiou C, Georgellis C, Rikas P, Anagnostou T. Renal parenchyma perforation and hematoma formation following double-J stent insertion in a solitary functioning kidney: an unusual complication. Case Rep Urol 2012;2012:301275. 23082274 10.1155/2012/301275 PMC3469072

[3] Akkaya Z , Oksuz N, Coruh AG. A rare complication of a common procedure: undiagnosed subcapsular renal hematoma after double-J stent insertion. Int J Case Rep Imag 2014;5:76–9.

[4] Aljuhayman A , Balaraj F, Ghazwani Y, Hamri SB. Subcapsular double-J stent following ureteroscopy: unique complication. J Surg Case Rep 2020;2020:rjaa404. 33101643 10.1093/jscr/rjaa404 PMC7569744

[5] Pradhan A , Rana YP. An uncommon situation of kidney perforation with a ureteric stent. Central Eur J Urol 2014;67:113–4. 10.5173/ceju.2014.01.art28 PMC4074716 24982799

